# Prevalence and clinical significance of cardiovascular magnetic resonance adenosine stress-induced myocardial perfusion defect in hypertrophic cardiomyopathy

**DOI:** 10.1186/s12968-020-00623-1

**Published:** 2020-05-04

**Authors:** Eun Kyoung Kim, Sang-Chol Lee, Sung-A Chang, Shin-Yi Jang, Sung Mok Kim, Sung-Ji Park, Jin-Oh Choi, Seung Woo Park, Eun-Seok Jeon, Yeon Hyeon Choe

**Affiliations:** 1 Division of Cardiology, Department of Medicine, Samsung Medical Center, Heart Vascular Stroke Institute, Sungkyunkwan University School of Medicine, #81 Irwon-ro, Gangnam-gu, Seoul, 06351 South Korea; 2 Department of Radiology and Cardiovascular Imaging Center, Samsung Medical Center, Sungkyunkwan University School of Medicine, 50 Irwon-ro, Gangnam-gu, Seoul, 06351 South Korea

**Keywords:** Cardiomyopathy, hypertrophic, Magnetic resonance imaging, Myocardial perfusion imaging, Myocardial ischemia, Microcirculation

## Abstract

**Background:**

Hypertrophic cardiomyopathy (HCM) is thought to be associated with microvascular dysfunction. Adenosine stress-perfusion cardiovascular magnetic resonance imaging (CMR) is a sensitive method for assessing microvascular perfusion abnormalities. We evaluated the prevalence and clinical characteristics of HCM patients with adenosine-induced perfusion defects on CMR.

**Methods:**

Among 189 consecutive patients with HCM who underwent adenosine-stress perfusion CMR, 115 patients who had clinical, echocardiography, 24-h Holter monitoring and treadmill exercise test data were analyzed. We calculated myocardial perfusion ratio index from the intensity-over-time curve to quantify perfusion defects. The presence and extent of the stress-induced perfusion defect were compared with clinical characteristics, presence and extent of late gadolinium enhancement (LGE), left ventricular (LV) mass index and volume, presence of non-sustained ventricular tachycardia (NSVT) and results of treadmill exercise test.

**Results:**

The mean age of enrolled patients was 51.8 ± 11.3 years. Most patients were asymptomatic except 25 subjects presented with New York Heart Association Class II dyspnea and 16 patients with atypical non-exertional chest discomfort. LGE was present in 103 (89.6%) subjects. Adenosine stress-induced perfusion defects were present in 48 (42%) subjects. None of the perfusion defects corresponded with a single or multiple coronary artery territories, showing a multiple patchy pattern in 24 (50.0%), a concentric subendocardial pattern in 20 subjects (41.7%), and as a single blot-like defect in the remaining 4 (8.3%). A perfusion defect was associated with NSVT, LV apical aneurysm, higher LV mass index, and higher LGE volume on univariate analysis. Multivariate analysis revealed female gender (*P* = 0.008), presence of apical aneurysm and NSVT (*P* = 0.036 and 0.047, respectively), and LV mass index (*P* = 0.022) to be independently associated with adenosine stress-induced perfusion defects.

**Conclusions:**

In patients with HCM, adenosine-stress perfusion defects on CMR are present in more than 40% of subjects. This perfusion defect is associated with NSVT, higher LV mass index, and apical aneurysms. The prognostic value of this finding needs further elucidation.

## Background

Hypertrophic cardiomyopathy (HCM) is a primarily myocardial disease with inappropriate growth and disarray of myocardial cells as well as interstitial fibrosis [1]. Since its original description, HCM has also been thought to be associated with myocardial ischemia due to microvascular dysfunction [2–5]. Nevertheless, in vivo studies of myocardial perfusion abnormalities in HCM are rare [6–8].

Cardiovascular magnetic resonance (CMR) has generated a great interest for assessing the myocardial abnormalities in HCM, especially for the presence of fibrosis in the hypertrophied myocardium with late gadolinium enhancement (LGE) in the myocardium [9–14]. Adenosine-stress perfusion CMR is a highly sensitive method for assessing myocardial perfusion abnormalities [15–17], and it has also been found to be useful for detecting perfusion abnormalities in suspected coronary artery disease as well as with microvascular dysfunction [18].

Our study sought to evaluate the frequency of microvascular perfusion abnormality in HCM assessed by adenosine-stress perfusion CMR and to elucidate its relationship with clinical characteristics as well as risk factors associated with HCM.

## Methods

### Study population

We prospectively enrolled 189 consecutive subjects diagnosed with HCM between December 2008 and November 2012 who underwent CMR with adenosine stress perfusion study that were included in the HCM registry of our cardiovascular imaging center as part of a research protocol. The diagnosis of HCM was based on the conventional guideline [19]; echocardiographic evidence of significant left ventricular (LV) hypertrophy which was defined as LV wall thickness of ≥15 mm in the absence of any other significant cardiac or systemic disease that could lead to concentric LV hypertrophy. Exclusion criteria were: Presence of cardiac or systemic disease causing concentric hypertrophy or increased wall thickness (e.g. aortic stenosis, amyloidosis, or systemic hypertension); age < 18 or > 75; typical chest pain of angina pectoris and documented coronary artery disease; symptoms of moderate to severe heart failure (New York Heart Association (NYHA) functional class III or IV); known ischemic coronary disease; and contraindications for CMR; Subjects whose exercise electrocardiogram (ECG) results showed significant ST segment changes (down-sloping or horizontal ST segment depression of more than 1 mm, ST segment elevation, or development of left bundle branch block) were excluded from the analysis. The study protocol was reviewed and approved by the institutional ethics review board of our institute.

### Clinical information

Clinical information including history of syncope or heart failure, and family (defined as first-degree relative) history of HCM or sudden cardiac death were collected, as well as cardiac risk factors. Twenty-four-hour ECG monitoring and symptom-limited treadmill exercise stress test were performed within a month of the CMR in subjects who were available for the tests. Results of the treadmill exercise test analyzed were; duration of exercise, adequacy of blood pressure response to exercise (defined as blood pressure increase of 20 mmHg or more), and presence of significant ST segment changes during exercise. The presence of LV outflow tract obstruction was assessed by echocardiography, with a characteristic late peaking velocity of > 3 m/sec as the definition of flow obstruction at rest or with Valsalva maneuver.

### CMR protocol

CMR was performed using a 1.5 T CMR system (Avanto B15, Siemens Healthineers, Erlangen, Germany) with a standardized CMR protocol with subjects in the supine position using a 32-element-phased-array cardiac coil. In detail, breath-held cine-CMR images of the whole heart were obtained with a ECG-gated balanced steady-state free precession sequence (echo time/repetition time/flip angle 1.31 ms/3.09 ms/72°, spatial resolution 1.72 × 1.29 × 6 mm^3^, 30 heart phases) to evaluate the LV function and myocardial mass. After the 2-chamber, 3-chamber, and 4-chamber long-axis views were obtained, short-axis stack cine-CMR images were obtained to cover the whole LV from the apex to the base with a slice thickness of 6 mm with 4 mm gaps. Stress perfusion CMR was performed with a 3-min continuous intravenous infusion of adenosine with a rate of 140 μg/kg/min. After the 3-min infusion, 0.1 mmol/kg of gadobutrol (Gadovist, Bayer Healthcare, Berlin, Germany) was injected as a bolus application with a rate of 3.0 ml/sec followed by a 20 ml saline flush. Four short-axis slices were obtained with TurboFLASH sequence without breath-hold for 1 min and the adenosine infusion was terminated subsequently. Fifteen minutes after termination of the adenosine infusion, an identical perfusion sequence was applied at rest. LGE images were obtained 10 min after the second contrast injection using a 2-dimensional TurboFLASH phase-sensitive inversion-recovery sequence (echo time/repetition time/flip angle 3.6 ms/8.14 ms/25°, spatial resolution 2.08 × 1.56 × 6 mm^3^, slice thickness 6 mm, inversion time adjusted for each patient to obtained optimal nulling of normal myocardium) in short-and long-axis planes.

### CMR analysis

CMR scans were visually interpreted by two experienced readers who were blinded to the clinical and laboratory data. In case of disagreement, a third experienced reader confirmed the findings. The types of HCM were categorized as septal, apical, septal and apical, concentric, and other. The LV end-diastolic and end-systolic volumes, ejection fraction, and mass were analyzed using commercialized software (CASS MRV CMR analysis software Version 3.3, Pie Medical Imaging B.V., Maastricht, Netherlands) by manually tracing the endocardial and epicardial contours in the short-axis stacks of end-diastolic and end-systolic images. For perfusion analysis, stress and rest perfusion scans were magnified and displayed and read simultaneously. The presence of perfusion defect was defined by regional hypoenhancements that persist for more than 10 frames after maximal myocardial enhancement [20]. Perfusion analysis of each myocardial segment was performed using the 16-segment model excluding the apical cap (modified from the 17-segment model recommended by the American Society of Echocardiography) [21]. The perfusion defects were also analyzed for correspondence with a coronary artery territory (left anterior descending coronary artery: anterior or septal segments together with apical segments; left circumflex coronary artery: lateral segments; right coronary artery: inferior segments), and categorized as non-corresponding when the sites of the defect were dispersed without correspondence to any one or multiple coronary artery territories. For quantitative analysis of the perfusion defects, the 16 segments were individually analyzed using the same software as above by modification of previously described methods [22, 23]. In brief, after the intensity-over-time curves were generated for each segment and the LV cavity blood pool in each corresponding slice, the maximum upslope (intensity/sec) of the myocardium were normalized to the LV cavity upslope to obtain myocardial perfusion ratio index (MPRI) for each segment. The MPRI were averaged for all myocardial segments. Myocardial LGE analysis was also performed using the 16-segment model with a cut-off value of signal intensities of over 6 standard deviations (SD) compared to the normal myocardial segment, as signal intensity of 6 SD or more above remote myocardium has been shown to have the best accuracy to demonstrate histology-verified fibrosis in HCM and enables comparisons with recent major studies [4, 24–27]. When the sites that showed perfusion defects at stress overlapped with the sites with LGE, the perfusion defects were considered to be caused by the fibrotic tissue at the site, and thus rendered stress negative. When LGE was present, the boundaries of the sites showing LGE were manually traced and the volume was calculated with the same software. The extent of LGE was quantified as a percentage of the total LV myocardial volume. The presence of an LV apical aneurysm was also assessed and expressed as present or absent.

### Evaluation of epicardial coronary arteries

Structural stenosis of the epicardial coronary arteries were evaluated in a proportion among the subjects in the stress CMR positive groups who agreed to receive the tests. Invasive coronary angiography was performed in 3 and coronary computed tomography angiography was performed in 21 subjects. All of the studies revealed normal or unremarkable findings of less than 10% diameter epicardial coronary artery stenosis.

### Statistical analysis

The subjects were divided into two groups: stress CMR positive and negative. All analyses were performed with PASW statistics 18 (Statistical Package for the Social Sciences, International Business Machines, Inc., Armonk, New York, USA). The inter-observer agreement analysis between the original two readers was performed by obtaining the intraclass correlation coefficient (ICC, average and 95% confidence interval) by reliability analysis. Chi-square association analysis was performed for evaluation of association of the stress-induced perfusion defects and the collected clinical information, type of HCM, the presence of LGE, and presence of apical aneurysm. Association between the stress-induced perfusion defect and LV volume, LV ejection fraction (LVEF), LV mass index, LGE volume, and LGE percentage was evaluated by the Mann-Whitney analysis. Multivariate analysis was performed with binary logistic regression analysis for evaluation of independent association of factors. Data are represented in frequency or mean ± standard deviation, and a *P* value of less than 0.05 was considered significant.

## Results

### Clinical characteristics

After exclusion of subjects according to the exclusion criteria, a total of 115 subjects were included in our analysis. Their mean age was 51.8 ± 11.3 years and male was predominant (83.5%). A history of diabetes was present in 4 (3.5%), current smoking in 30 (26.1%), and 28 (24.3%) were ex-smokers (defined as abstinence from smoking for at least 1 year). Twenty-five subjects presented with NYHA Class II dyspnea (21.7%), 16 presented with atypical non-exertional chest discomfort (13.9%), and the remaining were asymptomatic. History of syncope was present in 9 (7.8%), and family history of HCM and sudden cardiac death was present in 8 (7.0%) and 13 (11.3%), respectively.

### Laboratory and echocardiographic findings

Non-sustained ventricular tachycardia (NSVT) was detected in 11 (10.7%) of the 103 subjects who received 24-h ECG monitoring. Treadmill exercise stress test was performed in 102 subjects, and the mean duration of exercise was 9.4 ± 2.3 min. Inadequate blood pressure response to exercise was detected in 24 subjects (23.5%).

HCM was of septal type in 40 (34.8%), septal/apical type in 32 (27.8%), apical type in 28 (24.3%), concentric type in 13 (11.3%), and others in 2 (1.7%). LV outflow obstruction with systolic anterior motion of the mitral valve on echocardiography was present in 31 (27.0%) subjects.

### CMR findings

None of the patients had an abnormal blood pressure response to adenosine (Table 1). None experienced substantial vasodilator or gadolinium-related adverse symptoms except mild chest discomfort (20.9% of all patients) during the infusion of adenosine. Mean LV end-diastolic and end-systolic volume, LVEF, and LV mass index were 138.9 ± 27.1 ml, 39.4 ± 12.4 ml, 71.6 ± 7.3%, and 88.2 ± 29.9 g/m^2^, respectively. LGE was present in 103 (89.6%) subjects, and mean LGE volume and percentage were 20.0 ± 20.5 ml and 11.6 ± 8.9%, respectively. An apical aneurysm was found in 13 (11.3%) subjects.

**Table 1:** Hemodynamic response and safety data during cardiovascular magnetic resonance (CMR) examination

	*N* = 115
Before adenosine infusion	
Systolic blood pressure, mmHg	148 ± 18
Diastolic blood pressure, mmHg	76 ± 12
Heart rate, beats per minute	76 ± 67
During adenosine infusion	
Systolic blood pressure, mmHg	147 ± 19
Diastolic blood pressure, mmHg	75 ± 12
Heart rate, beats per minute	77 ± 13
After CMR examination	
Systolic blood pressure, mmHg	144 ± 21
Diastolic blood pressure, mmHg	72 ± 12
Heart rate, beats per minute	83 ± 14
Chest discomfort during infusion, n (%)	24 (20.9%)

**Fig. 1:**
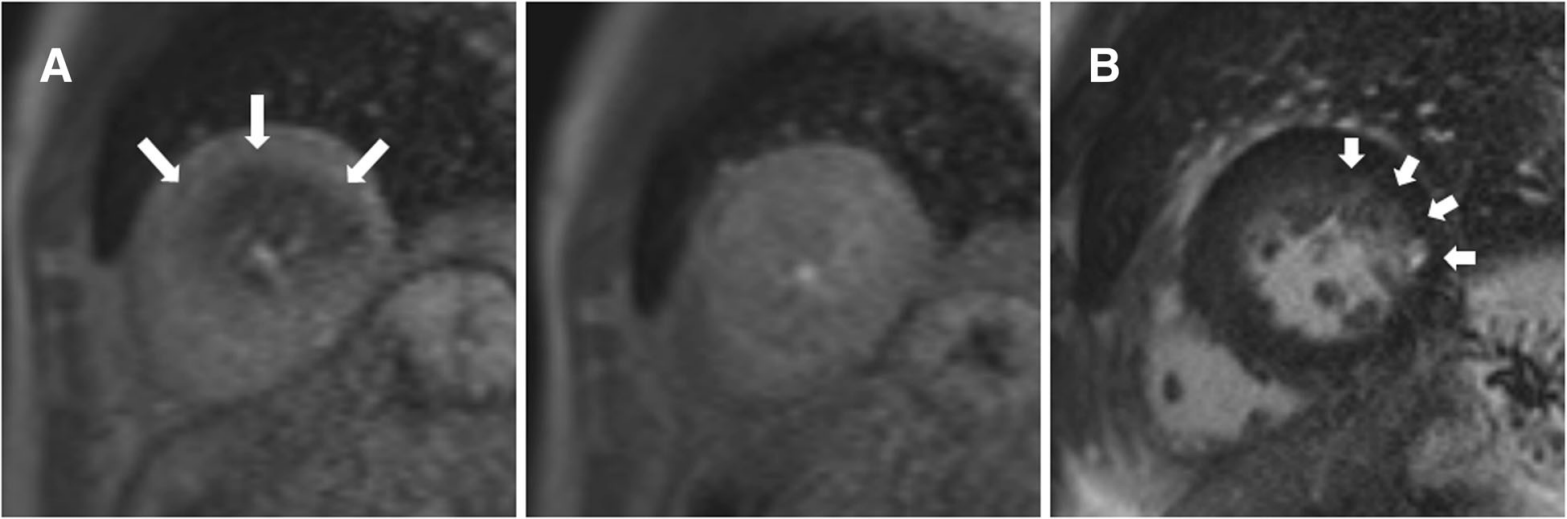
Patchy adenosine stress-induced perfusion defects on cardiovascular magnetic resonance (CMR) and late gadolinium enhancement (LGE). **a.** Short-axis adenosine-stress perfusion images of the left ventricle at stress (left) and rest (right) show patchy reversible perfusion defects (large arrows) at the junction between midventricular and apical segments. **b.** LGE analysis by inversion recovery show LGE (small arrows) in midventricular segments that are separate from the stress perfusion image slices

**Fig. 2:**
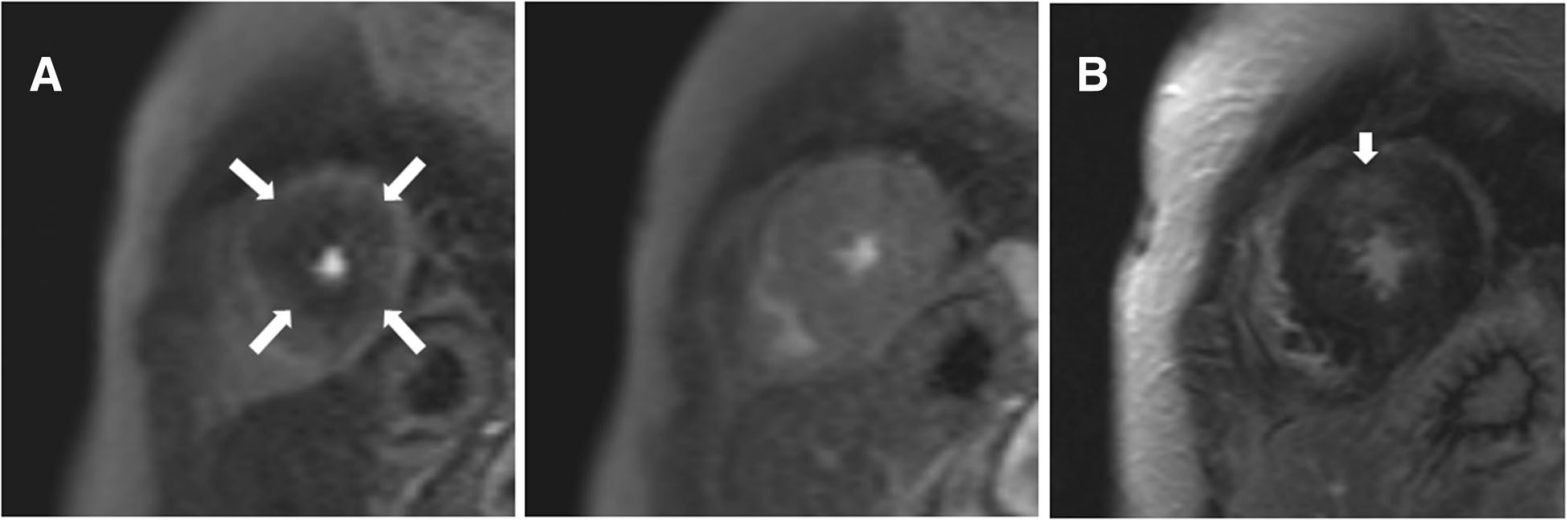
Concentric adenosine stress-induced perfusion defect on CMR and LGE. **a.** Short-axis adenosine-stress perfusion images of the left ventricle at stress (left) and rest (right) show concentric reversible perfusion defects (large arrows) at the apical segments. **b.** LGE analysis by inversion recovery show focal LGE (small arrows) in the anterior apical segment. Most of the area with concentric perfusion defect shown on stress-perfusion images do not overlap with the LGE area

**Fig. 3:**
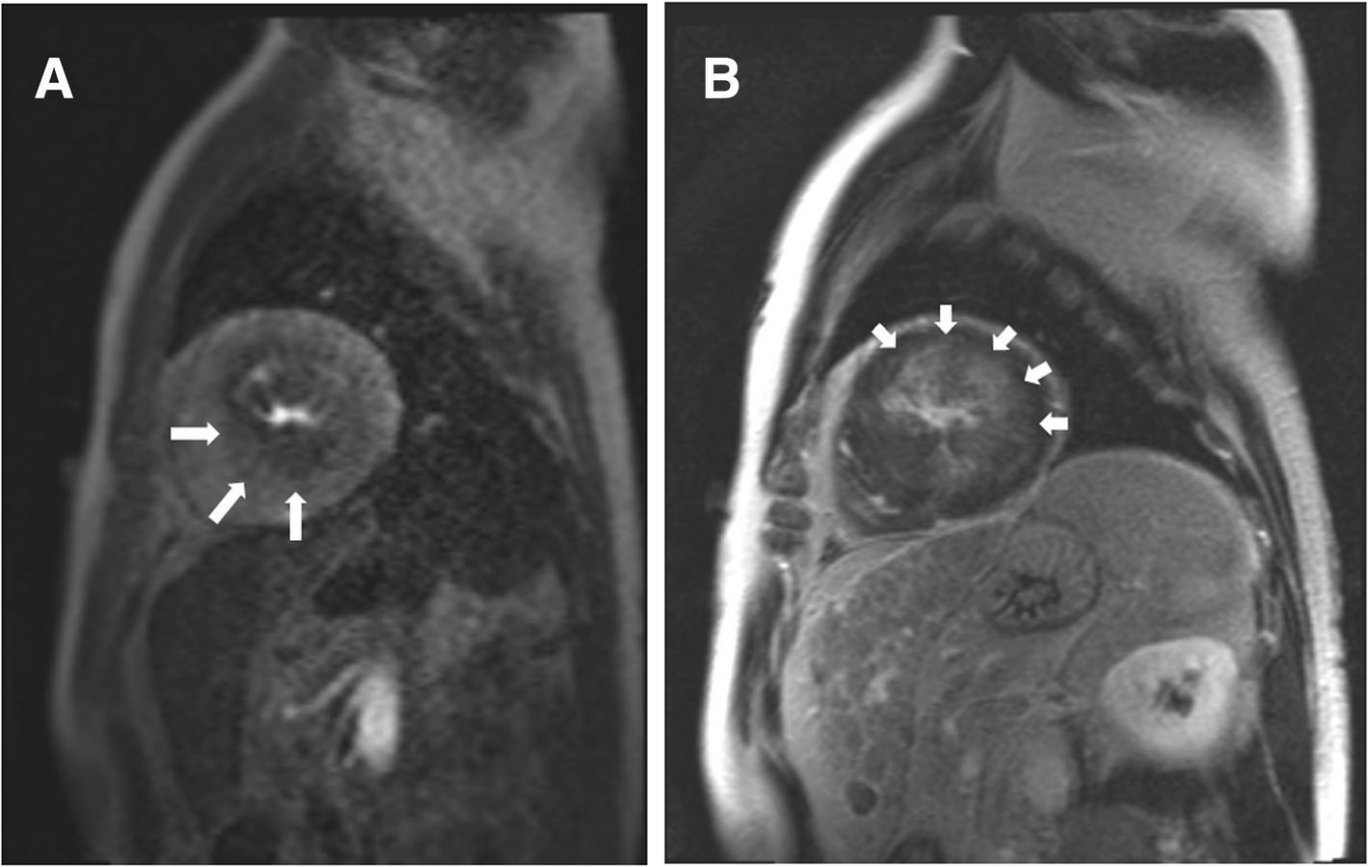
Focal blot-like adenosine stress-induced perfusion defect on CMR and LGE. **a.** Short-axis adenosine-stress perfusion images of the left ventricle at stress show concentric reversible perfusion defects (large arrows) at the septal and inferior midventricular segments. **b.** LGE analysis by inversion recovery show patchy LGE (small arrows) in the anterior and lateral midventricular segments. Most of the area with focal perfusion defect shown on stress-perfusion images do not overlap with LGE area

Visually assessed stress-induced perfusion defects were present in 48 (41.7%) patients. None of the defects corresponded with a single or multiple coronary artery territories, showing a multiple patchy pattern in 24 (50.0%), a concentric subendocardial pattern in 20 subjects (41.7%), and as a single blot-like defect in the remaining 4 (8.3%) (Figs. 1, 2 and 3). The ICC for the visual assessment of stress-induced perfusion defects between the original two readers was 0.94 with a 95% confidence interval from 0.90 to 0.96. On quantitative analysis, mildly significant difference in MPRI was found between the stress-positive group and the stress-negative group (0.83 ± 0.19 vs. 0.94 ± 0.30, *p* = 0.046). However, when the stress-negative group was divided into two groups (no perfusion defect group and perfusion-defect-corresponding-to-LGE-site group), significant differences were found between the no perfusion defect group and the perfusion-defect-corresponding-to-LGE-site group (1.12 ± 0.46 vs. 0.87 ± 0.15, *p* < 0.01), and also between the stress-positive group and the no perfusion defect group (*p* = 0.001). There was no significant difference in MPRI between the stress-positive group and the perfusion-defect-corresponding-to-LGE-site group.

### Difference in clinical and laboratory characteristics between stress-positive and negative groups

There was no difference in age, gender ratio, frequency of history of diabetes, and frequency of current or ex-smokers between the stress-positive and the stress-negative groups. History of syncope and family history of sudden cardiac death or HCM were not different between the two groups either. Symptoms of dyspnea and atypical non-exertional chest pain were also not associated with positive results on stress perfusion (Table 2).

**Table 2:** Comparison of clinical characteristics between CMR adenosine stress-positive and negative groups

Variables	Stress positive (*n* = 48)	Stress negative (*n* = 67)	*P* value
Age in years	50.6 ± 11.6	52.6 ± 11.1	0.34
Male (%)	37 (77.1)	59 (88.1)	0.12
Diabetes (%)	2 (4.2)	2 (3.0)	0.73
Atypical chest pain (%)	5 (10.4)	11(16.4)	0.36
NYHA class II dyspnea (%)	13 (27.1)	12 (17.9)	0.24
History of syncope (%)	3 (6.3)	6 (9.0)	0.60
Family history of			
Sudden cardiac death (%)	5 (10.4)	8 (11.9)	0.80
HCM (%)	3 (6.3)	5 (7.5)	0.80
Smoking			0.70
Current (%)	14 (29.2)	16 (23.9)	
Ex-smoker (%)	10 (20.8)	18 (26.9)	

*NYHA* New York Heart Association, *HCM* Hypertrophic cardiomyopathy

The type of HCM, exercise duration on treadmill exercise test, and frequency of LV outflow tract obstruction were not associated with the presence of stress-induced perfusion defects. NSVT on 24-h ECG monitoring was found more frequently in the stress positive group (*p* = 0.027), but exercise duration on treadmill test or the frequency of abnormal blood pressure response to exercise were not different between the two groups (Table 3).

**Table 3:** Comparison of Echocardiography and Other Lab Findings Between CMR Adenosine Stress-Positive and Negative Groups

	Stress positive (*n* = 48)	Stress negative (*n* = 67)	*P* value
Type of HCM			0.33
Septal (%)	17 (35.4)	23 (34.3)	
Apical (%)	9 (18.8)	19 (28.4)	
Septal+Apical (%)	14 (29.2)	18 (26.9)	
Concentric (%)	8 (16.7)	5 (7.5)	
Mixed (%)	0 (0)	2 (3.0)	
LV outflow obstruction (%)	15 (31.3)	16 (23.9)	0.38
NSVT on 24-h holter monitoring (%)	8/43 (18.6)	3/60 (5.0)	0.03
Inadequate blood pressure response to exercise (%)	12/45 (26.7)	12/57 (21.1)	0.51
Exercise duration on stress treadmill test (min:sec)	9:18 ± 2:21	9:51 ± 2:34	0.30

*HCM* Hypertrophic cardiomyopathy, *LV* Left ventricular; *NSVT* Nonsustained ventricular tachycardia

**Fig. 4:**
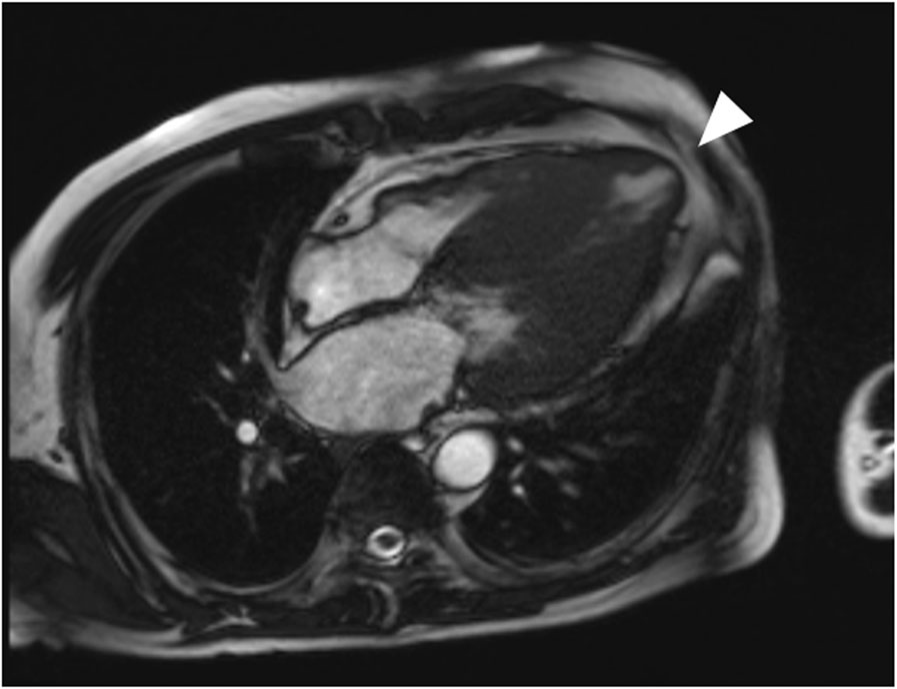
Apical aneurysm on 4-chamber long axis cine CMR (arrowhead) detected on the same patient on Fig. 3

Data from CMR are summarized in Table 4. Neither the LV volumes nor the LVEF showed any significant difference between the two groups. Mean LV mass index was significantly greater in the stress-positive group (98.0 ± 30.9 vs. 81.3 ± 27.3 g/m^2^, *p* = 0.003). The presence or absence of LGE was not significantly different between the two groups, with 45 subjects (93.8%) in the stress-positive group and 58 (86.6%) subjects in the stress-negative group showing LGE. However, the presence of stress-induced perfusion defects was significantly associated with higher LGE volume and also with LGE volume percentage (26.0 ± 24.7 vs. 15.6 ± 15.6 ml, *p* = 0.012, 14.1 ± 9.9 vs. 9.9 ± 7.6%, *p* = 0.015, respectively). Apical aneurysm on CMR (Fig. 4) was found more frequently in the stress positive group (10 vs. 3 subjects, *p* = 0.006).

**Table 4:** Comparison of CMR parameters between adenosine stress-positive and negative groups

Variables	Stress positive (*n* = 48)	Stress negative (*n* = 67)	*P* value
LVEF (%)	71.7 ± 7.4	71.5 ± 7.3	0.34
LV EDV (ml)	139.7 ± 23.7	138.3 ± 29.5	0.78
LV ESV (ml)	39.4 ± 11.5	39.5 ± 13.1	0.99
LV mass index (g/m^2^)	98.0 ± 30.9	81.2 ± 27.3	0.003
LGE (+) case, n	45 (93.8%)	58 (86.6%)	0.21
LGE volume (ml)	26.0 ± 24.7	15.6 ± 15.6	0.007
LGE percentage (%)	14.1 ± 9.9	9.9 ± 7.6	0.011
Apical aneurysm, n	10 (20.8%)	3 (4.5%)	0.006

*LVEF* Left ventricular ejection fraction, *EDV* End-diastolic volume, *ESV* End-systolic volume, *LGE* Late gadolinium enhancement

Multivariate regression analysis revealed female gender (odds ratio [OR] with 95% CI 8.33 [1.72–33.33], *p* = 0.008), presence of apical aneurysm (5.58 [1.12–27.7], *p* = 0.036), NSVT on 24-h ECG (6.38 [1.03–39.63], *p* = 0.047), and higher LV mass index (1.03 [1.00–1.06], *p* = 0.022) to be independently associated with presence of stress-induced perfusion defects (Table 5).

**Table 5:** Multivariate analysis of clinical and CMR findings between the stress-positive and negative groups

Variables	OR (95% Confidence Interval)	***P*** value
Age	0.98 (0.95–1.01)	0.233
Gender, male vs. female	0.12 (0.03–0.58)	0.008
History of diabetes	1.90 (0.15–20.6)	0.598
History of smoking	0.21	
Ex-smoker	1.23 (0.36–4.22)	0.740
Current smoker	0.75 (0.21–2.71)	0.657
Atypical chest pain	0.30 (0.06–1.53)	0.148
History of syncope	1.95 (0.29–12.9)	0.490
NSVT on 24-h holter	6.38 (1.03–39.6)	0.047
Inadequate blood pressure response to exercise	0.66 (0.18–2.36)	0.523
LV outflow tract obstruction	0.32 (0.08–1.26)	0.102
Presence of apical aneurysm	5.58 (1.12–27.7)	0.036
LGE volume	0.99 (0.97–1.03)	0.946
LV mass index	1.03 (1.01–1.06)	0.022

*OR* Odds ratio. *P* value were calculated with multiple logistic regression

*LV* Left ventricular, *NSVT* Non-sustained ventricular tachycardia, *LGE* Late gadolinium enhancement

## Discussion

This study sought to assess the prevalence and possible clinical implications of stress-induced myocardial perfusion defects on CMR in subjects with HCM. According to our results, stress-induced myocardial perfusion defects which reflect myocardial ischemia in the hypertrophied myocardium was a relatively frequent finding, and was associated with various significant findings in HCM such as NSVT on 24-h ECG monitoring, apical aneurysm, and increased LV mass index. These findings arouse suspicion that microvascular dysfunction leading to stress-induced perfusion defects on CMR are significantly related to consequences that may lead to patient deterioration and sudden cardiac death in HCM. To our knowledge, this is the largest study its kind, and may elucidate the importance of evaluating microvascular dysfunction in this patient population.

The significance of microvascular dysfunction in HCM has been a subject of interest since the early days of the description of the disease [2–4]. According to previous reports, microvascular dysfunction and resulting myocardial ischemia may be of utmost importance in understanding the natural course of the disease and risk stratification for high-risk subjects for sudden cardiac death [4]. It has also been noted that assessment of myocardial ischemia may be crucial in evaluating subjects with HCM [6]. A recent study reported that microvasculopathy may be an intrinsic feature of HCM with similar characteristics across the natural phases of the disease, and suggested that this may induce prolonged intramyocardial ischemia resulting in extensive scar formation and consequent end-stage evolution [28]. However, few investigations have been performed in this area and stress-induced myocardial perfusion defects have not been established as a possible clinical risk factor or target of management. Furthermore, even fewer reports have been published on the prevalence of myocardial ischemia or stress-induced perfusion defects in HCM [29, 30], and they have utilized nuclear imaging methods only.

CMR has been shown to be a highly sensitive method for assessing myocardial perfusion abnormalities not only in patients with coronary artery disease, but also in subjects with microvascular dysfunction [15, 16, 31, 32]. Utilization of this method in addition to plain LGE imaging in CMR may be a useful way to evaluate the microvascular dysfunction associated with HCM or other myopathic processes. There have been a few reports on stress-induced myocardial perfusion defects detected by CMR in patients with HCM [8, 33–35], but no large-scaled studies have demonstrated the clinical characteristics or significance of the finding in detail.

There have been previous reports associating microvascular dysfunction depicted by stress-induced myocardial perfusion abnormalities on nuclear studies in HCM with clinical deterioration or death [4, 36]. In agreement with prior investigations which showed reduced myocardial perfusion reserve in HCM being correlated with the magnitude of hypertrophy [4, 7], our data demonstrated a significant association between the stress-perfusion defect and LV mass index. In addition, NSVT on holter and apical aneurysm which were risk factors for poor prognosis in HCM were also associated with stress perfusion defect on CMR. The fact that NYHA class, exercise duration, or history of syncope did not show significant correlation with stress-perfusion defect in our data may have been due to the fact that the subjects enrolled in our study were mostly asymptomatic or had mild symptoms and the proportion of subjects with substantial symptoms were too small to show any significant results. Taken together, there have been notions that CMR stress perfusion analysis may have utility in predicting prognosis and risk stratification [37], but as the associations of stress-induced perfusion defects on CMR with the above findings do not directly indicate that the perfusion defect is a significant independent risk factor or prognostic indicator in HCM at this time, further prospective studies may be needed to evaluate the clinical value of stress-induced perfusion defects on CMR.

We found female patients had stress-perfusion defects more frequently. Although not proven in any large-scale studies, microvascular dysfunction without significant epicardial coronary artery disease is thought to be more prevalent in women [38, 39], but this is the first time it has been demonstrated in HCM subjects. However, as the number and proportion of women in this study are relatively small, further studies are needed to confirm this relationship. Meanwhile, as various previous reports indicate that women with HCM show a more adverse prognosis [40, 41], this may be an interesting subject to pursue in future studies.

In comparison with the previous studies, our patients showed a higher prevalence of LGE on CMR.The prevalence of myocardial LGE in patients with HCM has been reported to vary from 33 to 84% [42–44]. Those reports commented that considering the heterogeneity of inclusion criteria for patient groups and scar pattern in HCM, such a wide range of reported incidence was not surprising. In our study, we included LGE that show focal, patchy or diffuse patterns in the right ventricular insertion site of interventricular septum as well as the mid-ventricular and epicardial wall, and the presence of myocardial fibrosis may have shown to be more frequent compared to other studies which enrolled limited LGE patterns in the mid-ventricular and epicardial wall only. We also believe that the classification of small focal hyperenhancements as significant LGE may have resulted in a higher incidence of LGE compared to other studies.

**Fig. 5:**
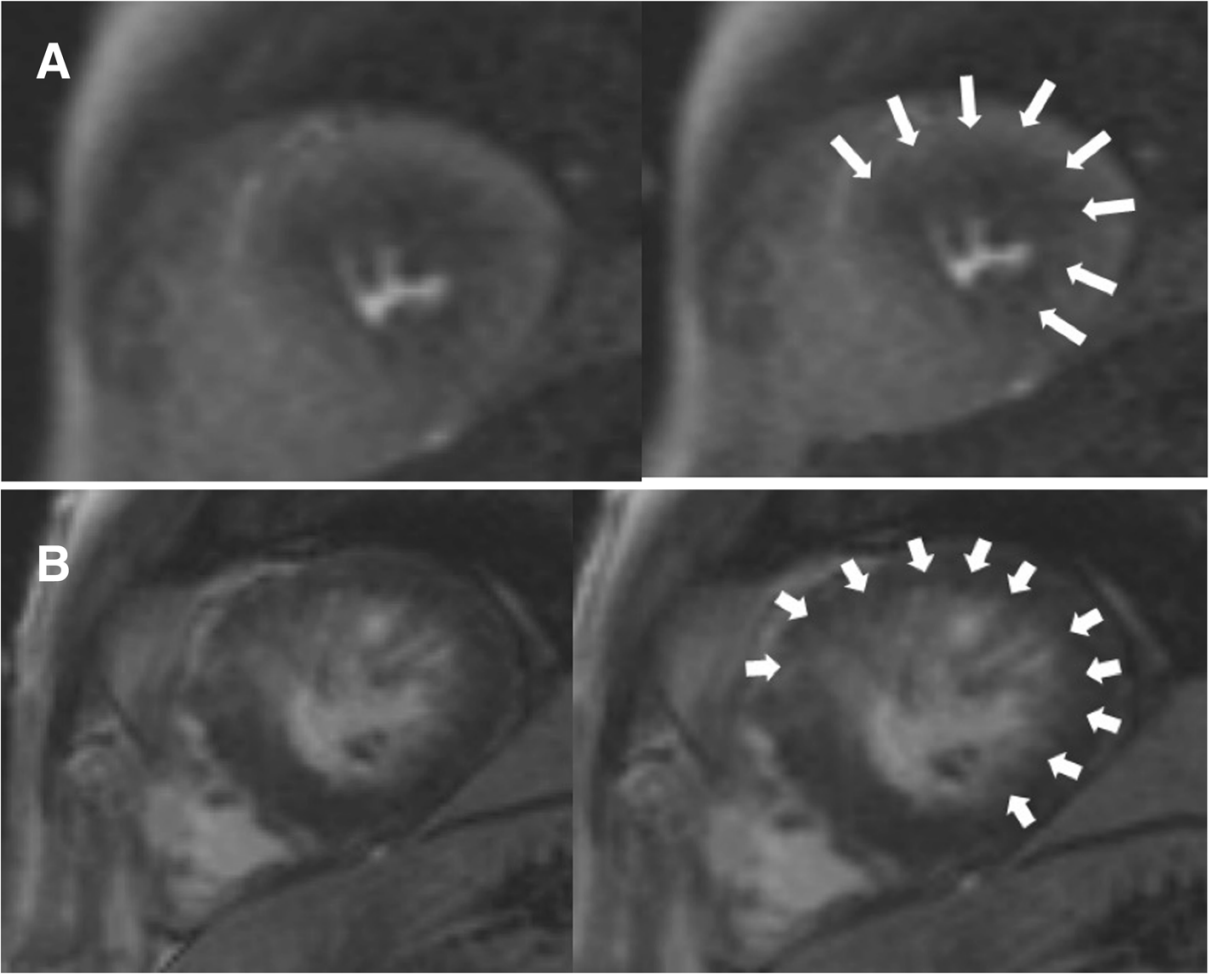
Example of adenosine-stress induced perfusion defect (**a**, long arrows) that overlapped with the site of LGE (**b**, short arrows). This subject was categorized as adenosine-stress CMR negative

Of note is caution in evaluating the stress-induced perfusion defects on CMR. As the stress perfusion test in CMR is performed before delayed image acquisition for LGE analysis, it is well known that the first-pass perfusion defect at the time of the initial contrast injection may also be related to the presence of massive fibrosis of the lesion and may correspond to the site of LGE. As this is the case, we carefully omitted the lesions that overlapped with the LGE (Fig. 5) and only included the defects that were not related to the LGE site. As this was the case, the findings on the quantitative analysis of the stress-perfusion defects showing significant difference in MPRI between the stress-positive group and the no perfusion defect group should be acknowledged, leaving out the analysis performed for the perfusion-defect-corresponding-to-LGE-site group.

### Study limitations

Our study has some limitations. Firstly, only a proportion (31%) of the study subjects that showed perfusion defects on stress CMR received invasive coronary angiography or coronary computed tomography angiography within 3 months of the CMR, although all of them showed normal or insignificant results. Hence, the co-existence of epicardial coronary artery disease cannot be completely excluded. However, the stress-induced perfusion defects did not correspond to a coronary artery territory, and subjects who showed significant ECG changes on the symptom-limited treadmill exercise ECG tests performed in about 90% of the subjects were all excluded. Furthermore, coronary artery disease cannot account for the perfusion defect rate of 40% in Korean patients with a mean age of 50. Secondly, 24-h ECG monitoring and treadmill exercise ECG were performed in most, but not in all subjects. However, subjects who did not undergo these tests were very few, and the proportion of the subjects who did not receive the test was similar between the stress-positive and negative groups, this limitation may not be critical to our analysis. Lastly, parametric mapping data could not be analyzed as the CMR in our study was performed before parametric mapping was incorporated into wide clinical use. Future studies using native T1 mapping and extracellular volume may be warranted to obtain additional information on the association between myocardial ischemia and LGE.

## Conclusions

Adenosine-stress-induced perfusion defects on CMR are frequently found in subjects with HCM. The finding is associated with increased LV mass index, presence of apical aneurysm, and inadequate hemodynamic response to exercise. The clinical significance and prognostic value of the finding need further elucidation.

## Data Availability

The datasets used and/or analyzed during the current study are available from the corresponding author on reasonable request.

## Abbreviations

CMR: Cardiovascular magnetic resonance
ECG: Electrocardiogram
EDV: End-diastolic volume
ESV: End-systolic volume
HCM: Hypertrophic cardiomyopathy
LGE: Late gadolinium enhancement
LV: Left ventricle/left ventricular
LVEF: Left ventricular ejection fraction
LVOT: Left ventricular outflow tract
MPRI: Myocardial perfusion ratio index
NSVT: Non-sustained ventricular tachycardia
NYHA: New York Heart Association
